# Ethnicity, social connectedness, and the rural-urban food continuum: Food security among urban informal settlement dwellers in Kenya

**DOI:** 10.1016/j.heliyon.2024.e30481

**Published:** 2024-04-29

**Authors:** Oscar Ingasia Ayuya

**Affiliations:** Department of Agricultural Economics and Agribusiness Management, Egerton University, P.O Box 536-20115, Egerton, Kenya

**Keywords:** Food security, Social connectedness, Rural-urban food continuum, Informal settlement, Food deserts

## Abstract

Rapid urbanization in developing countries is increasingly becoming an important development issue due to its negative effects on poverty and food insecurity in cities. This study investigated the determinants of the food insecurity gap among urban households living in informal settlements in Nairobi, Kenya, and the role of social connectedness and the rural-urban food continuum. Using panel data collected from 385 households using a two-stage cluster sample design, the study applied panel data regression and decomposition models to understand the factors affecting food insecurity access scores and weekly household food consumption expenditure. Descriptive results showed a score of 8.00 for the pooled sample on the household food access scale, with households from other regions having a lower score (7.94) than those from the Western region (8.32). 43 % of households from Western Kenya engaged in farming in rural areas (43 %) and had higher social connectedness (45 %) than households from other regions. The results indicate that dependency ratio, income, savings, social connectedness, rural visits, and dietary knowledge are significant factors impacting food security. Decomposition of levels and change in food insecurity revealed that endowments from the random effects model contributed to reducing the food insecurity gap between households originating from the Western region and those from other regions. The study highlights the importance of considering regional factors and promoting social connectedness and rural-urban linkages in addressing food insecurity in informal urban settlements.

## 1 Introduction

Urbanization is increasingly becoming an important development issue for developed and developing countries [1] (Kuddus et al., 2020). The urban population is projected to rise to 4.98 billion in 2030 from 2.86 billion people in 2000 to 4.5 billion in 2022 [2,3] (Cohen, 2004; Macrotrends, 2024). Recent estimates indicate that about 1.1 billion people live in informal settlements globally, representing an increase of 165 million people in the last two decades [4] (Reckford & Aki-Sawyerr, 2023). People living in informal settlements are often poor, with inadequate access to healthy food, housing, and sanitary services [5,6] (Szabo, 2015; Aboulnaga et al., 2021). Specifically, informal settlements in less developed countries have metamorphosed into food deserts, with households facing significant challenges in access to safe, affordable, and quality food [7,8] (Battersby & Crush, 2016; Crush et al., 2018). This amplifies pre-existing urban inequalities, exacerbates food insecurity, and overburdens vulnerable slum dwellers with malnutrition and health problems. The intricate connection between the growth of informal settlements and food insecurity in urban areas calls for a holistic strategy that confronts this food security problem as a threat to achieving sustainable development goal (SDG) 2 targets.

Urban food insecurity is a serious challenge in sub-Saharan Africa. About 15 % of the urban population in the region is food insecure [9] (WFP, 2022). The rapid urbanization, economic crises, and the recent coronavirus pandemic further exacerbate this issue. About 238 million people in sub-Saharan Africa lived in informal settlements [10] (United Nations, 2023). The region also has the largest proportion (56 %) of the urban population living in informal settlements [11] (Zerbo et al., 2020). Although food insecurity in informal settlements varies across countries, the average food poverty in most countries in sub-Saharan Africa is higher than their peers in Asia and Latin America. For instance, 85 % and 74 % of slum dwellers in Nairobi and Addis Ababa are malnutrition [11] (Zerbo et al., 2022). Additionally, another study indicated that 6 % and 13 % of the children in informal settlements in Kenya were wasted and underweight, respectively [12] (De Vita et al., 2019). Battersby and Watson [7] attribute food insecurity in urban informal settlements to poverty, unemployment, and inefficient food urban food systems.

Food deserts are burgeoning and garnering attention in Kenya's food landscape because the country hosts Kibera – one of the largest informal settlements in Africa [13] (Soma et al., 2022). The food security problem in Kibera is attributed to poverty, poor infrastructure, unemployment, high food prices and limited access to essential services [14,15] (Gallaher et al., 2013; Ayuya et al., 2021). These factors prevent households in Kibera from adequately accessing and consuming nutritious food. Mutisya et al. [16] and Cardosi et al. [17] also observed that food insecurity in Kibera is exacerbated by ethnic makeup and the intricate social connections in the informal settlement. Additionally, food security challenges in urban areas emerge from converting agricultural land in urban peripheries to commercial and residential settlements [18] (Huho & Muriuki, 2021). Thus, the interplay of economic, infrastructural, and social factors and the prevalence of food insecurity highlight the need for a more nuanced understanding of the food insecurity landscape in Kibera to contribute to finding lasting solutions.

Several studies have investigated urban food security in Kenya, primarily concentrated on rural food insecurity, while the distinct challenges urban informal settlements face have received less attention. Studies that have focused on urban food security have either relied on cross-sectional data to disentangle the problem [13,14] (e.g., Gallaher et al., 2013; Soma et al., 2022) or not recognized the interplay of social or cultural connectedness and role of rural-urban food systems in explaining gaps in food security outcomes among urban populations living on informal settlements [[19], [20], [21]] (e.g., Korir et al., 2022; Kimani-Murage et al., 2014; Omondi et al., 2017). Furthermore, although studies several studies in Kenya have investigated the potential role of rural-urban food continuum in enhancing food security in urban areas [[22], [23], [24]] (Onyango et al., 2021; Merchant et al., 2022; Onyango et al., 2023), they overlook region of origin of urban populations and do not employ the decomposition analysis method to explore the differential impacts of rural-urban food continuum and social connectedness on urban food systems outcomes.

This research aims to bridge these gaps by delving into the intricacies of food insecurity gaps in Kibera, explicitly focusing on the rural-urban food continuum and the significance of sociocultural connectedness – social capital and networks based on region of origin and ethnicity. The uniqueness of our study stems from the decomposition of the food insecurity gap between urban households originating from different regions in the country in a panel setup. This study aims to inform more effective interventions and policies by providing a deeper understanding of the issue.

The study findings are central to formulating urban food security solutions considering informal settlements' social dynamics. The study also showcases the potential role of the rural-urban food continuum in combating the food insecurity menace in informal settlements in urban areas. A rural-urban food continuum can enhance access to diverse and nutritious food options, contributing to improving food and nutrition security and creating economic opportunities for residents. The study also contributes to understanding social capital and networks in shaping urban food security. Therefore, the study contributes to the increasing recognition of the prevalence and complexity of food insecurity by uncovering the underlying factors beyond the informal nature of settlements.

The paper is organized as follows: The next section provides a conceptual framework that underpins the study based on literature; the third section is the methodology that describes the study area, methods, and empirical model. The results and discussion section provides statistical and contextual interpretation of the results, while the conclusion section summarizes the results and implications of the study.

## 2 Conceptual approach

The food security analysis in Kibera is premised on a conceptual framework adapted from Ericksen [25] conceptualization of food systems. Ericksen defined food security as the interactions between and within biogeophysical and human environments, food production and consumption activities, food systems outcomes and determinants. Food production activities are undertaken to produce food. Examples of food production activities are the acquisition and ownership of land and inputs for crop production. Other activities are processing and packaging of food, distribution, and retailing. Consumption is a downstream activity that involves decisions on which food to acquire, prepare, and consume at the individual or household level. One of the outcomes of food systems is food security, which has three components: access, availability, and utilization. The three components of food security are influenced by production, distribution, and exchange (availability), affordability, allocation, and preferences (accessibility), nutritional value, social value, and food safety (utilization) [25].

The study acknowledges determinants of food security outcomes as espoused by Erickson in the formulation of the conceptual framework of this study. The underpinning of the conceptual framework is the ethnic makeup of Kibera district. Although non-natives, Luo and Luhya ethnic groups from Western Kenya account for 63 % of the population in the Kibera [26] (Marx et al., 2019). Although ethnically distinct, the Luhya and Luo tribes share similarities, including economic systems and food culture. For instance, both tribes have diets heavily based on agricultural produce and rely on social ties during food crises to smooth consumption. Our conceptual framework also acknowledges that food systems are closely linked to socio-cultural systems [27] (Opiyo & Agong, 2020) of these two tribes and account for the possible influence of food culture, customs, preferences, and social ties in food consumption.

Recent literature on urban food security recognizes the importance of the rural-urban food continuum in enhancing food security. Onyango et al. [22] report that over 50 % of households across Nairobi city, including Kibera, received food remittances from rural areas. Most households surveyed received food remittances were born in rural areas [22], underlining the role of connection to rural households in food security. About 80 % of households receiving food transfers from rural areas received it from relatives, with some receiving food from friends in rural areas [2]. Some households also received food from friends and relatives in urban areas. However, in another study, Onyango et al. [24] also focused on the length of stay in the city, rural-urban links, and food transfers, which did not focus on the dietary diversity of households in Nairobi. Nonetheless, the results reported by Onyango et al. [22] are further supported by Merchant et al. [23], who found that urban households reported lower food security outcomes than rural households because of decreased access to the food production environment resulting from COVID-19 restrictions on movement and lockdowns.

Ericksen [25] also identifies other factors, such as access to government and non-government social support services, as food production and consumption determinants. Besides the rural-urban food continuum, employment, education, household income, and food prices also determine food security outcomes [23,24]. These studies highlight the importance of socioeconomic variables in determining food security. The study acknowledged the food security challenges experienced by households in the study areas and conceived a set of variables ranging from food production to acquisition and consumption. The study considered two variables to capture rural-urban linkages – farming in rural areas and visits to rural areas. These two variables capture the movement of people and food between rural and urban areas and demonstrate urban households’ social connections to the rural areas, which can enhance food security in urban regions [26] (Frayne et al., 2004). Farming and visits to rural areas also demonstrate rural-urban linkages as survival strategies for the poor in informal settlements. The strong linkages could involve food transfers contributing to urban food security [27] (Lesetedi et al., 2003).

The study also adapts [28] Serageldin and Grootaert (2017) definition of social capital as networks, norms, and social trust that facilitate coordination and cooperation for mutual benefit. It uses this definition to consider neighbours of the same culture and whether households stayed in the informal settlement since birth as a measure of social capital. The study uses living in the same location since birth to reflect possible deep-rooted social networks that result from long residency that create trust, reciprocity, and mutual aid among neighbours. The study also conceive that having neighbours of the same culture breeds shared understanding, social cohesion, and the willingness of people from the same background to support one another.

Furthermore, following Ericksen's [25] conceptualization of determinants of food security outcome included households' access to government food transfer programs and NGO support as important determinants of food security in informal settlements. The study conceptualized that socioeconomic variables such as income and investment, employment, and age of respondents are important determinants of food security [23,24] (Merchant et al., 2022; Onyango et al., 2003). The study uses the dependency ratio as a proxy for the economic well-being of the household as it reflects economically productive members of the household [29] (Iram & Butt, 2004). The study considered having savings, investment, and access to loans as safety nets, economic buffers, and resilience to crises [30] (Prosekov & Ivanova, 2018), contributing to food security. These variables, alongside households' access to government and non-government social support services, social capital variables, and rural-urban food continuum variables, were used to influence food security in Kibera.

The study also posited that nutritional knowledge can also impact food security. According to Grunert et al. [31] and Lombe et al. [32], nutrition knowledge is positively associated with food security. Therefore, dietary knowledge was incorporated as an index of urban households' knowledge about nutrition. According to Ericksen [25], food security was defined as having access to food. The study chose accessibility over availability because, according to Burfeind [33], there is an over-emphasis on food availability in both literature on food security and policy focus.

## 3 Methodology

### 3.1 Study area

The study was conducted in Kibera district in Nairobi County of Kenya. The district is a densely populated informal settlement covering approximately 2.1 square kilometers, and an informal settlement is one of the largest urban slums in Africa [34,35] (Anele, 2021; KNBS, 2019). Its total population is estimated at 120,057 people, distributed in about 39,593 households [35] (KNBS, 2019). The slum has inadequate housing, electricity, water and a sewerage system [15] (Ayuya et al., 2021). It is overcrowded, and most residents are food insecure [20,36] (Kimani-Murage et al., 2014; Kirui et al., 2022). A significant proportion of Kibera's residents live in makeshift houses constructed from mud, timber, and corrugated metal sheets [34] (Anele, 2021). Most of the Kibera population is unemployed and has food insecurity [20] (Kimani-Murage et al., 2014). The settlement is characterized by a diverse population, with residents from various regions and ethnic backgrounds [16,17]. However, the dominant ethnic communities are Luo (36 %), Luhya (27 %), and Kamba (15 %) [26]. Therefore, Kibera's unique context justifies situating this study to understand the determinants of the food security situation and identify potential interventions to improve the food security situation in the informal settlements in Kenya.

### 3.2 Data

**Fig. 1:**
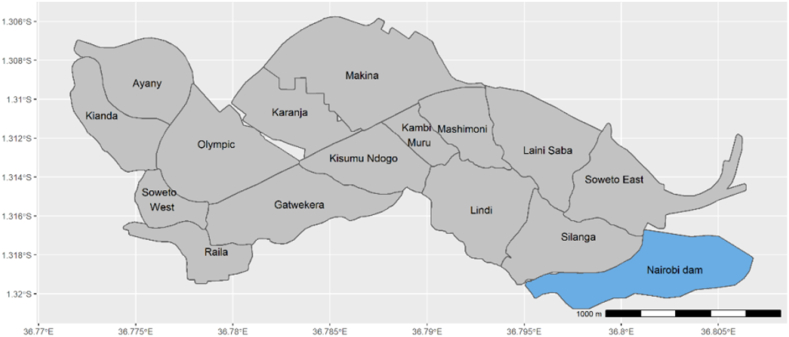
Map of the study area.

The study baseline data 385 households in Kibera in 2020. The first survey was conducted in August 2020 and collected data from households in 15 villages in the Kibera villages. The data collection activity was part of the Feeding Cities and Migration Settlement project, a research project that aimed to contribute to sustainable, resilient, and urban food systems in Kenya's urban area [37] (Soma, 2020). A sampling procedure for an unknown population was used to determine the sample size. The housing and population census data does not provide information on the region of origin for urban households. Fig. 1 shows a map of the study area.

A two-stage cluster sample design was applied to select villages and households. In the first stage, 14 out of 15 villages of Kibera were selected, with the thirteenth village excluded due to security concerns. The remaining twelve villages were allocated equal samples. In the second stage, a random walk technique that relied on Personal Digital Assistants (PDAs) was used to select respondents from the twelve villages randomly. Field supervisors identified significant landmarks in mapping the households, such as markets, schools, and religious sites per village cluster. A script determined the random walk's starting point, direction, and sampling interval. The random walk resulted in a sample size of 385 farmers for 2020. In August 2021, a follow-up survey was conducted, revisiting 308 of the initial 385 households.

**Fig. 2:**
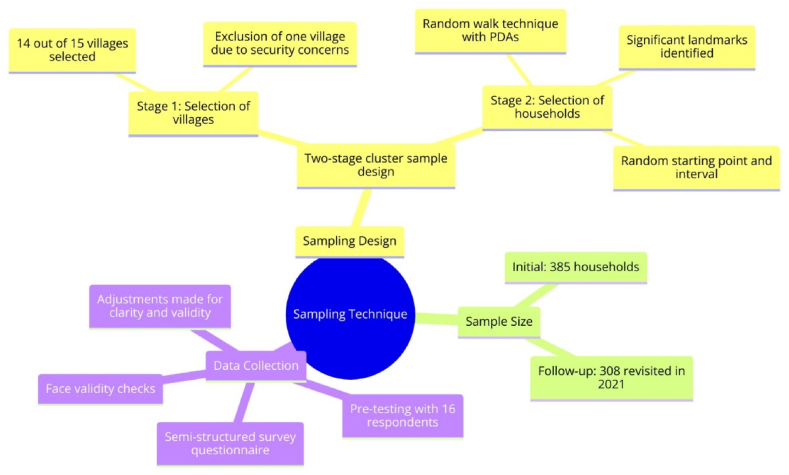
Sampling and data collection technique.

Data were collected from the selected household using a semi-structured survey questionnaire. The data collection tool was pre-tested with 16 respondents at baseline to identify and rectify ambiguous questions, test the clarity of the questions, assess relevant questions, and determine the appropriate survey length. The piloting of the survey tool was also crucial in checking the technical functionality of the digital tool and helped in refining data collection procedures. Coupled with face validity checks, necessary adjustments were made to the survey tool after the piloting, which ensured its validity and reliability. The sampling procedure is presented in Fig. 2.

Information collected from households using the semi-structured survey included socioeconomic factors such as income, education, age, gender, household size, savings, and marital status. Other information collected included the regional background of households, social capital and networks, rural connections, rural and urban farming, and access to external support (e.g., government subsidy and support from NGOs). The Household Food Insecurity Access Scale (HFIAS) was used to measure food (in)security. The HFIAS questions were adapted to the local context, resulting in eight questions instead of the standard nine questions. Thus, the HFIAS score ranged between 0 and 24, with a higher score indicating higher food insecurity. The HFIAS food security categories were generated as follows: Food secure: Score = 0, Mildly food secure (score = 1–13), Moderately (score = 14–16), and severely food insecure (Score = 17–24) [38] (Coates et al., 2007).

Ethical issues were considered in the study as the study involves human participants. Approval was provided by the Wageningen Economic Research (WUR) Research Ethics Committee (REC). The REC approved the project proposal based on the reviews of its ethical safeguards. Additionally, relevant research approvals were sought from the National Commission for Science, Technology, and Innovation (NACOSTI) to conduct the study in Kenya. All respondents were given verbal consent at the start of the survey. Additionally, all the research assistants involved in the study were trained on ethical procedures in data collection, including confidentiality and cultural sensitivity.

### 3.3 Empirical model

Kroger and Hartmann's [39] extension of the Kitagawa-Oaxaca-Blinder decomposition approach in panel data was used to decompose disparities in food (in)security between urban households originating in Western Kenya and other regions. Let *Y*_*it*_ denote the food insecurity access score for household *i* at time *t*, *X*_it_ represents a vector of explanatory variables, including socioeconomic variables, rural-urban food continuum, social capital and networks, and external support, β a vector of coefficients of the respective independent variables, α signifies the intercept term, μ_i_ and ν_it_ denote time-invariant time-varying components of the error term. Thus, the panel random effects model to for food (in)security was specified as follows:

1 $$Y_{\text{it}}=\alpha +X_{\text{it}}\beta +\mu_{i}+\nu_{\text{it}}$$

First, the study estimated separate random effects regression equations for urban households from the Western region and urban households from other regions, and then, it used a complete model that combined the two types of households. Next, the study used the Kroger and Hartmann [39] approach; the study used the Kitagawa-Oaxaca-Blinder decomposition to break down the food insecurity gap between the two groups into endowments (differences in characteristics), coefficients (differences in the impacts of characteristics), and random effects (unexplained discrepancies) contributions.

The Kitagawa-Oaxaca-Blinder decomposition of the food (in)security was expressed as:

2 ΔY=ΔX*βW+Δβ*∂O+Δμ

where Δ*Y* denotes the food insecurity gap between the urban households from the western region (W) and urban households from other regions (*O*); *ΔX* represents the difference in average characteristics between the two groups; βW denotes coefficients of characteristics for urban households originating from Western region; *∂O* is the average characteristics of the urban households originating from other regions; *Δβ* signifies the difference in coefficients between the two groups; and *Δμ* refers to the unexplained differences.

The study estimated the contributions of endowments (the observed characteristics), coefficients (the impact/relationship endowments and outcome variable), and random effects (unexplained part) to the food insecurity gap. It assessed the impact of various household characteristics on food insecurity access for the two groups. The decomposition of the food security scores provided detailed information on the contributions of endowments, coefficients, and random effects to the food insecurity gap between the two categories of urban households.

Panel data analysis relies on several assumptions to produce unbiased results. The study deliberately exploited a broad range of options provided by Kroger and Hartmann [39] extension for specifying the model. Adjustment to various factors in model specification and decomposition was conducted to handle serial correlation, heteroskedasticity, cross-sectional dependence, and endogeneity. The study introduced a longitudinal weights option in the structure of the model to allow for a more accurate estimation of the coefficients and endowments. This accounted for the potential bias from panel dropout. The study used the factor variable prefix in the model (name) option to ensure that the fundamental functional form of the model was correctly specified. These options addressed serial correlation concerns while maintaining the integrity of the analysis. Furthermore, Kroger and Hartmann [39] extension of the Kitagawa-Oaxaca-Blinder decomposition approach in panel data is flexible regarding modelling time effects, group effects, and non-linear time trends. Therefore, the application of the model accounted for unobserved heterogeneity. Lastly, the study employed bootstrapping to bolster the model's robustness against autocorrelation and heteroskedasticity.

## 4 Results

### 4.1 Descriptive results

Table 1 presents descriptive findings of household demographics, social capital, rural-urban continuum, and external support by region of origin for participants. Most households across the two panels were male-headed, with household heads being 42 years of age. Significantly higher proportions of households originating from Western were male-headed and had younger household heads than those originating from other regions. The average dependency ratio was 46, with households from Western Kenya having a significantly higher dependency ratio (48 %) than those from other regions (48 %). 19 % of surveyed households had lived in the exact location since birth. A significantly higher proportion of households from other regions (34 %) compared to the Western region (2 %) reported living in the same location since birth. About 45 % of the households had people from their culture as neighbours, with a notably greater proportion of those hailing from the Western region (47 %) than households from other regions (43 %).

**Table 1:** Descriptive results of household demographics, social capital, rural-urban continuum, and external support by region of origin.

	Pooled		Other		Western		
Variable	Mean	SD	Mean	SD	Mean	SD	Difference
Male-headed HH	0.70	0.46	0.66	0.47	0.73	0.44	−0.07^b^
Age of household head	41.73	12.61	42.66	13.33	40.72	11.71	1.93^b^
Dependency ratio	45.65	23.89	43.49	24.34	48.02	23.20	−4.54^b^
Log monthly income	9.06	1.25	9.05	1.31	9.07	1.19	−0.02
Savings	0.16	0.37	0.17	0.38	0.15	0.35	0.03
Loan	0.37	0.48	0.38	0.49	0.37	0.48	0.01
Investment	0.14	0.35	0.15	0.36	0.13	0.34	0.02
Same location since birth (%)	0.19	0.39	0.34	0.48	0.02	0.14	0.32^c^
% same culture	44.88	25.80	42.96	26.38	46.98	25.03	−4.03^b^
Farming in rural areas	0.21	0.41	0.18	0.39	0.24	0.43	−0.06^a^
Visits to rural areas	1.49	1.73	1.48	2.09	1.51	1.20	−0.04
Government subsidy	0.21	0.41	0.21	0.41	0.20	0.40	0.01
NGO support	0.49	0.50	0.49	0.50	0.50	0.50	0.00
DKI	30.62	3.43	30.80	3.30	30.41	3.55	0.39
HFIAS	8.12	4.63	7.94	4.79	8.32	4.44	0.39
Food expenditure	12,575	10,045	12,915	11,115	12,204	8729	711

***Note:*** Dietary knowledge index (DKI).

a *p-value < 0.1.*

b *p-value < 0.05.*

c *p-value < 0.01*.

Approximately 21 % of surveyed households engage in rural farming. A marginally significant difference exists between households from the Western region (24 %) and those from other regions (18 %). The average number of visits to rural areas per year is 1.49, with no significant differences between households from Western Kenya (1.51) and those from other regions (1.49). In addition, 21 % and 49 % of households received government and non-governmental food subsidies and provisions, respectively. No notable differences were observed between the two farmer groups regarding receiving support from government and non-governmental organizations for food supply.

With a mean HFIAS score of 8.12 for the pooled sample, households in the Western region (8.32) might experience slightly more food insecurity than those from other regions (7.94). Nonetheless, the difference of 0.39 between the two regions lacks statistical significance, implying that the discrepancy might be due to random variation rather than a genuine disparity in food security between the regions. The pooled sample's average weekly food expenditure is 12,575. Although households from other regions (KES 12,915) spent more on food than those from the Western region (KES 12,204), with a difference of 711, this distinction is not statistically significant. Like the HFIAS score, the KES 711 difference in weekly expenditure between the two household groups could be due to random variation instead of a genuine difference in food spending between them.

Table 2 presents the prevalence of food insecurity across time by origin of households in Kibera. The results are based on household food insecurity access prevalence (HFIAP). This measure is appropriate when the studied population is expected to have a low or moderate prevalence of food insecurity [35,38]. Unlike the HFIAS score, which was not statically significant, a comparison of the two groups revealed marginally statistically significant (p < 0.01) differences in the prevalence of food insecurity across time by region of origin of households between the two groups.

**Table 2:** Prevalence (%) of food insecurity across time by region of origin of households.

Category	Pooled	Other regions	Western	p-value
Food secure	9.52	11.33	7.55	0.067
Mildly food insecure	10.1	12.15	7.85	
Moderately food insecure	36.36	34.53	38.37	
Severely food insecure	44.01	41.99	46.22	

About 10 % of the sample households were food secure, with a slightly higher percentage among households from other regions (11 %) than those from the Western region (8 %). Further, 10 % of the pooled sample was mildly food insecure, with a higher percentage for households from other regions (12 %) than those from the Western region (8 %). Moderate food insecurity was reported by about 37 % of households, with households originating from Western Kenya (38 %) being moderately food insecure compared to 35 % of households from other regions in the country (Table 2). Severely food insecure households were 44 % of the total households surveyed. A higher percentage of households from the Western region (46 %) than those from other regions (42 %) were severely food insecure.

### 4.2 Econometric results

Table 3 presents the results of a full and reduced random effects model of determinants of food security access scores. The following interpretations of significant variables can be made based on the coefficients and their associated significance levels in the panel data analysis results. The dependency ratio had a positive effect on household food insecurity access score across all models, with coefficients of 0.027 (p < 0.01) in the full model, 0.031 (p < 0.01) in the other regions model, and 0.028 (p < 0.01) in the Western model. This means it was positively associated with food insecurity for full models and reduced models. The log monthly income had a negative effect on the household food insecurity access score. The coefficients for this variable are −0.544 (p < 0.01) for the full model, −0.669 (p < 0.01) for urban households from other regions, and −0.445 (p < 0.05) for households from the Western region. A 1 % increase in monthly income was associated with 54.4 %, 66.9 %, and 44.5 % decrease in household food insecurity for the full, other regions, and Western regions models, respectively.

**Table 3:** Random effects estimates of household food insecurity access score by origin of households.

	Full model		Other regions		Western regions	
Variable	Coeff.	SE	Coeff.	SE	Coeff.	SE
Farming in rural areas	−0.251	0.425	0.480	0.724	−0.688	0.523
Origin (0 = Other, 1 = Western)	0.178	0.361				
Dependency ratio	0.027^c^	0.007	0.031^c^	0.010	0.028^c^	0.010
Log monthly income	−0.544^c^	0.169	−0.669^c^	0.241	−0.445^b^	0.225
Savings	−2.635^c^	0.411	−2.428^c^	0.600	−2.835^c^	0.585
Loan	−0.114	0.355	0.386	0.480	−0.682	0.512
Investment	0.351	0.506	0.848	0.757	−0.507	0.693
HH has stayed in the location since birth	−0.424	0.520	0.084	0.551	−4.369^c^	1.181
% of neighbours of the same culture	−0.019^c^	0.006	−0.031^c^	0.009	0.001	0.009
Number of visits to rural areas	−0.239^a^	0.123	−0.221	0.145	−0.366^b^	0.208
Government subsidy	−0.202	0.396	−0.398	0.567	−0.130	0.578
NGO support	0.457^c^	0.327	0.460	0.497	0.358	0.469
Dietary knowledge index	−0.092^c^	0.044	−0.041	0.071	−0.112**	0.057
Year	0.645	0.310	0.297	0.444	0.640	0.466
Wald $\chi^{2}$	132.11^c^		78.81^c^		86.75^c^	
Rho	0.239		0.282		0.319	

a p-value <0.1.

b p-value <0.05.

c p-value <0.01.

Savings negatively impacted household food insecurity access score with coefficients of −2.635 (p < 0.01) in the full model, −2.428 (p < 0.01) in the other regions model and −2.835 (p < 0.01) in the Western model. For every shilling increase in savings, the household food insecurity access score decreased by 2.635 units in the full model, 2.428 units in the other regions model, and 2.835 units in the Western model. The percentage of neighbours from the same culture had negative effect on household food insecurity access score in the full model and for other regions model, with coefficients of −0.019 (p < 0.01) and −0.031 (p < 0.01), respectively. Specifically, an increase e in the percentage of neighbours from the same culture by 1 % decreased the household food insecurity access score by 0.019 units in the full model and 0.031 units in the other regions' model.

The number of visits to rural areas negatively affected household food insecurity in the full model and the Western model, with coefficients of −0.239 (p < 0.1) and −0.366 (p < 0.05), respectively. For every additional visit to rural areas, household food insecurity access score decreases by 0.239 units in the full model and 0.366 units in the Western model. NGO support positively affects household food security access score in the full model with a coefficient of 0.457 (p < 0.01). Households that received NGO support had a 0.457 food insecurity score higher than households non-recipients of NGO support. The dietary knowledge index negatively impacted the household food insecurity access score, with coefficients of −0.092 (p < 0.01) in the full model and −0.112 (p < 0.05) in the Western model. For every one-unit increase in dietary knowledge, the household food insecurity access score decreased by 0.092 units in the full model and 0.112 units in the Western model.

Additionally, the study conducted a random effects model analysis using weekly food consumption expenditure as the dependent variable to corroborate the results presented in Table 3. The food consumption estimates are presented in Table 4. To derive a conclusion regarding cross-validation based on the weekly household food consumption expenditure and food insecurity access score results, the study compared significant variables in both sets of results and evaluate how they align with each other. The weekly household food consumption expenditure results yielded significant variables, including farming in rural areas, dependency ratio, log monthly income, savings, investment, percentage of neighbours of the same culture, number of visits to rural areas, and NGO support. Meanwhile, food insecurity access score results (see Table 3 above) identified significant variables, including dependency ratio, log monthly income, savings, household staying in location since birth, percentage of neighbours of the same culture, number of visits to rural areas, NGO support, and dietary knowledge index.

**Table 4:** Random effects estimate of food consumption expenditure by origin of households.

	Full model		Other regions		Western Region	
Variable	Coeff.	SE	Coeff.	SE	Coeff.	SE
Farming in rural areas	0.177^c^	0.057	0.177^b^	0.089	0.220^c^	0.078
Origin (0 = Other, 1 = Western)	0.037	0.052				
Dependency ratio	0.001	0.001	0.003^b^	0.001	0.001	0.001
Log monthly income	0.067^c^	0.017	0.092^c^	0.023	0.054^b^	0.022
Savings	0.195^c^	0.061	0.156^a^	0.094	0.230^c^	0.081
Loan	0.062	0.048	0.116	0.073	0.020	0.071
Investment	0.161^c^	0.057	0.215^b^	0.094	0.118	0.075
HH stayed in the location since birth	0.039	0.072	0.066	0.078	0.197	0.169
% of neighbours of the same culture	0.002^a^	0.001	0.003^c^	0.001	0.001	0.001
Number of visits to rural areas	0.031^b^	0.016	0.029	0.020	0.025	0.028
Government subsidy	−0.024	0.054	−0.030	0.082	−0.037	0.070
NGO support	−0.085^a^	0.047	−0.171^b^	0.071	0.021	0.067
Dietary knowledge index	−0.008	0.007	−0.013	0.010	−0.003	0.010
Year	−0.023	0.038	−0.050	0.060	0.034	0.058
Wald $\chi^{2}$	65.51^c^		53.13^c^		33.98^c^	
Rho	0.465		0.437		0.507	

a p-value <0.1.

b p-value <0.05.

c p-value <0.01.

### 4.3 Decomposition of household food insecurity

Table 5 displays the households' average Household Food Insecurity Access Scale (HFIAS) scores over time, disaggregated by region of origin. HFIAS scores range from 0 to 24, where a higher score implies increased food insecurity. In the pooled data, Western region households (8.32) faced marginally high food insecurity levels from 2020 to 2021 compared to households from other regions (7.94), with a gap of 0.387. Although this difference is relatively small, it suggests higher food insecurity on average for households in the Western region. Households from the Western region had an average HFIAS score of 8.25 compared to 7.76 for households originating from other regions in 2020. The 0.490 gap in food insecurity indicates a somewhat more significant disparity than the pooled data suggests. However, in 2021, the gap between the two groups diminishes, with Western region households scoring an average of 8.40 on HFIAS and households from other regions scoring 8.17. The gap is reduced to 0.234, which is smaller than the gaps in the pooled 2020 data.

**Table 5:** Average HFIAS scores over time by region of origin of households.

	Total	Other regions	Western	Gap
Pooled	8.12	7.94	8.32	0.387
	(4.63)	(4.79)	(4.44)	
2020	7.99	7.76	8.25	0.490
	(4.88)	(5.09)	(4.63)	
2021	8.29	8.17	8.40	0.234
	(4.29)	(4.37)	(4.21)	

Note: Standard deviation provided in parentheses; the score is out of 27 based on the frequency of difficulties in accessing food.

Table 5 results indicate that households in the Western region generally experience higher food insecurity levels than households in other regions. The gap between the two groups narrows over time, albeit under increased food insecurity, with the smallest gap occurring in 2021. The increase in HFIAS scores in 2021 might be attributable to the coronavirus pandemic and high food prices, exacerbating food insecurity for more households. Urban households from other regions seem more affected, possibly because they engage less in rural farming and exhibit lower social connectedness than those from Western Kenya.

Table 6 shows levels and change decompositions for 2020 and 2021. The observed row reveals the mean differences in household food insecurity access scores between the groups, computed non-parametrically from the available data. In 2020, a 0.49 difference existed between households originating from the Western region and those from other regions. This difference decreased to 0.234 in 2021, signifying a reduction of the food insecurity gap.

**Table 6:** Oaxaca-Blinder decomposition results of household food insecurity access score from panel data.

	year		Year	
	2020	2021	2020	2021
Observed	−0.49	−0.234	0	0.256
Decomposition				
Endowments	0.003	0.028	0	0.024**
Coefficients	−0.178	−0.178	0	0.000
Interaction	0.000	0.000	0	0.000
RE	0.042	0.160***	0	0.118
Total	−0.133	0.009	0	0.142
Decomposition %				
Endowments	−2.567	303.235***	.	17.081***
Coefficients	134.185	−1954.2***	.	0.000
Interaction	0.000	0.000	.	0.000
RE	−31.619	1751.028***	.	82.919***
Total	100	100	.	100

The decomposition of levels unravels the food insecurity between urban households from the Western region and those from other areas. The levels decomposition divides the gap into four segments: endowments, coefficients, interactions, and random effects (RE). In 2021, the endowment component's positive value (0.028) reveals that the social capital and rural continuum food characteristics of urban households from the Western region played a role in reducing the food insecurity divide. The significant 303 % percentage contribution emphasizes the importance of social capital and rural continuum food characteristics in closing the gap between both groups. The coefficients (−0.178) component remains stable, suggesting no change in the influence of characteristics on food insecurity scores between the groups in 2021. A negative percentage (1954 %) contribution implies that the coefficients would have expanded the food insecurity gap if considered in isolation. However, since endowments and RE have positive contributions, the overall effect is a decrease in the gap.

The interactions component of zero indicates no joint impact of endowments and coefficients on the food insecurity gap. The random effects (RE) value of 0.160 points to an unexplained contribution to the 2021 food insecurity gap reduction, with a 1751 % percentage contribution emphasizing this unexplained portion's substantial effect on the gap decrease between Western region urban households and those from other regions. In essence, the decomposition of levels reveals that the endowments and RE components positively contributed to reducing the food insecurity gap between the two groups in 2021, while the coefficients component remained unchanged and did not result in an overall increase in the gap due to the other components' positive contributions.

The decomposition of the change table displays the changes in the food insecurity gap between urban households originating from the Western region and those from other regions during 2020 and 2021. The non-parametric alteration (0.256) shows a decrease in the food insecurity gap between the two groups from 2020 to 2021. The positive endowment value (0.024) indicates that household characteristics, including social capital and networks and the rural-urban food continuum, of urban households originating in the Western region contributed to closing the food insecurity gap between 2020 and 2021. The 17 % percentage contribution emphasizes the significance of these shifts in characteristics.

The zero value for the coefficient component suggests no change in the impact of characteristics on food insecurity scores between the two groups from 2020 to 2021, meaning the coefficient component did not contribute to the decrease in the food insecurity gap. The interactions component is zero, signifying no combined effect of endowments and coefficient changes on the food insecurity gap between 2020 and 2021. The positive RE value (0.118) shows an unexplained contribution to food insecurity gap reduction between 2020 and 2021, with an 83 % percentage contribution reflecting the unexplained portion's significant impact on the gap decrease between urban households from the Western region and other regions. In summary, the gap in food insecurity among the two groups lessened from 2020 to 2021, primarily because of the influences of the endowments and RE elements. The coefficients and interaction components, on the other hand, did not play a role in altering the food insecurity disparity.

## 5 Discussion

The results presented in Table 3 are consistent with previous studies that have examined the factors affecting food security in informal urban settlements and other contexts. Notably, the associations between dependency ratio, income, savings, social cohesion, rural visits, and dietary knowledge emerge as significant factors. The established positive relationship between dependency ratio and food insecurity is reiterated by earlier studies, indicating that households with a higher proportion of dependents are more susceptible to food insecurity due to increased resource pressure and limited income [[40], [41], [42], [43], [44]] (Aidoo et al., 2013; Ahmed & Abah, 2014; Agidew & Singh, 2018; Sisha, 2022; Dessie et al., 2022). Moreover, the negative correlation between monthly income and food insecurity has been documented in past research, suggesting that households with higher incomes enjoy better food security access, as they can afford a more diverse and nutritionally adequate diet [45,46] (Carson & Boege, 2020; Mei et al., 2020). Mei et al. [46], for example, uncovered a sequential relationship between income and household food security among migrant workers in Malaysia.

Table 3 also accentuates the significance of savings for food security. Households with more substantial savings can better withstand shocks, such as pandemics, unemployment, or illness, thereby maintaining food security [47]. Crucially, the study occurred during the height of the COVID-19 pandemic in 2020 and 2021, which disproportionately impacted households in informal settlements due to job and income losses. Resilience capacities, like savings, empower households to respond to crises [48], as demonstrated by COVID-19-related research. Laborde et al. [49] identified savings as a food insecurity mitigating factor for approximately 300,000 households worldwide. Households relying on personal savings during the pandemic were less likely to encounter food insecurity [50]. In Burkina Faso and Uganda, savings explained household adaptive capacities differences during the coronavirus pandemic [51,52] (Mahmud & Riley, 2021; Ouoba & Sawadogo, 2022). Consequently, household resilience capacities, such as savings, enable households to cope with and mitigate the impact of food insecurity, particularly during crises.

Table 3 results also underscore the importance of strong social networks and support systems, as exemplified by the percentage of neighbours sharing the same culture, in helping households manage food insecurity. Social capital may contribute to food security through the synergy created by the interrelationships among community members at every stage of the food system [53]. Additionally, the finding suggests that food-sharing practices, particularly those embedded in cultural and social traditions, may serve as a sustainable mechanism for coping with hunger. Social connectedness has also been linked to food security in urban settings, implying that better-connected communities are more likely to have access to sufficient food [54]. Thus, this finding suggests that social connectedness is associated with improved food access in urban areas, suggesting the need for policies and programs that promote social cohesion to address food insecurity.

Previous studies have also reported the association between visits to rural areas and better food security access. These studies posit that access to additional resources or support networks in rural areas can enhance food security for urban households [55]. Within the rural-urban food continuum framework, the results imply that increased exposure to rural areas, as denoted by the number of visits, correlates with lower levels of household food insecurity. This may suggest that households with frequent visits to rural areas maintain more robust ties to rural communities, which could influence their access to resources or networks that bolster food security among urban households.

The study confirms the importance of dietary knowledge in addressing food insecurity, corroborating earlier research linking nutrition education to households’ capacity to make healthier food choices and optimize limited resources to enhance food security. El Bilbeisi et al. [56] explored the relationship between household food insecurity and dietary intake and nutrition-related knowledge, attitudes, and practices in Palestine, finding that food-insecure households possessed inadequate nutrition-related knowledge and had unfavourable attitudes towards healthy eating. Conversely, El Bilbeisi et al. [56] reported associations between food attitudes, dietary habits and food security status among adults in households. Therefore, the study underscores the importance of dietary knowledge in addressing food insecurity, which aligns with prior research connecting nutrition education to healthier food choices and more efficient use of limited resources to improve food security.

The study reveals that the duration of residency in the same location was negatively associated with food insecurity for households originating from the Western region. There was a 4.7-unit reduction in food insecurity among households originating from western Kenya that had stayed in the same location since the formation of their households compared to those that migrated into the location. The results suggest long-term residency is linked to lower food insecurity access scores. The explanation for this association could be deep-rooted social connections created by longer stays in informal settlements that households rely on to access food during periods of food scarcity [22,30].

The study identified several consistent variables between both outcomes, including dependency ratio, log monthly income, savings, percentage of neighbours of the same culture, number of visits to rural areas, and NGO support. These overlapping significant variables imply coherence between factors that impact household food consumption expenditure and food insecurity access scores over time. A higher dependency ratio and NGO support were linked to increased food insecurity. In contrast, higher monthly income, savings, percentage of neighbours of the same culture, and the number of visits to rural areas were connected to lower food insecurity and elevated food consumption expenditure.

Nonetheless, unique variables emerged in each set of results, such as farming in rural areas, investment, household staying in location since birth, and dietary knowledge index, possibly indicating region-specific factors that influence food security. In the food consumption model, households engaged in rural farming spent 17.7 % more on food than those not involved in rural farming activities. The effect size of farming in rural areas on household weekly food consumption expenditure was more substantial for households originating from the Western region (22 %) than those from other regions (17.7 %). Concerning investment, the complete model result demonstrated that households with investments reported a 16.1 % surge in food consumption expenditure (p-value <0.01). However, investment was only significantly linked to consumption expenditure in the regression involving households from other regions. Households from other regions with investments increased food consumption by 21.5 % more than households without investments (p-value <0.05). This may be ascribed to factors such as financial security, wealth accumulation, and risk diversification resulting from investments that enable households to maintain or boost food consumption expenditure.

The positive association between farming in rural areas and household food expenditure reveals the potential role of the rural-urban continuum in enhancing food security in urban areas. Urban household farming in rural areas possibly accessed supplementary food resources or income stemming from their farming activities away from the city. The results reveal that the interconnectedness of rural and urban areas through agricultural activities is crucial for enhancing food security in urban areas, especially for poor and food-insecure households [57]. The flow of food from rural to urban areas enables households in urban areas to take advantage of the rural region's agricultural productivity and diversity to secure access to food. Policies and interventions that recognize and bolster these rural-urban connections can improve food security outcomes in urban areas.

Nonetheless, the observed variation in the effect size and significance levels between households from the Western region and those from other regions implies that the benefits derived from rural farming activities may not be uniform across different regions. Households from the Western region appear to benefit more from their rural farming engagement, possibly due to regional differences in agricultural productivity. This highlights the importance of considering regional factors when designing and implementing policies or interventions to improve food security in urban households through rural-urban linkages.

The cross-validation of outcomes from weekly household food consumption expenditure and food insecurity access score uncovers a significant overlap between the variables impacting food security. This suggests that these models offer a consistent comprehension of the factors influencing food security in the examined regions. The consistent variables can be dependable indicators for policy interventions addressing food security concerns. Nevertheless, it is crucial to recognize and consider the unique variables in each model, as they may represent household-specific factors that merit further investigation.

## 5. conclusion

This study contributes to understanding food security determinants in informal urban settlements, shedding light on the role of social connectedness and the rural-urban food continuum. The findings underscore the significance of dependency ratio, income, savings, social connectedness, rural visits, and dietary knowledge as factors affecting food security. The results suggest that interventions to promote social cohesion and foster rural-urban connections may be instrumental in enhancing food security among urban households.

The study found that the rural-urban food continuum, demonstrated by the link between countryside visits and farming in rural areas, underscores the value of fostering robust ties between city households and rural areas. This suggests that interventions promoting resource sharing and supporting rural-urban food systems could enhance urban household food security. This study also validates the significance of dietary knowledge in tackling food insecurity, supporting prior research linking nutrition education to households’ abilities to choose to optimize scarce resources for better food security. Consequently, interventions to improve food security should consider incorporating nutrition education components to empower urban households to make more informed food choices.

Social connectedness addressed the food insecurity challenge in the informal urban settlement. This insight implies that initiatives that strengthen social connectedness and community support systems may positively impact urban households' food security. Furthermore, the decomposition analyses highlight the need to consider regional factors and context-specific food security determinants when designing policies and interventions. The observed difference in rural farming activities' impact on food security among households from the Western region and those from other regions highlights the need for tailored approaches in addressing the diverse challenges urban households face. Considering regional differences and context-specific factors, the study findings offer essential insights for policymakers and practitioners seeking to develop targeted interventions to enhance food security in urban settings. There is a need for policy to emphasize the critical role of social connectedness and rural-urban linkages in enhancing food security among urban informal settlement dwellers. This would require interventions that support rural-urban food systems.

The limitation of the study is the geographical scope. The study was confined to Kibera Nairobi, limiting the generalizability of the findings to other urban informal settlements within Kenya. Future research should explore the dynamics of social capital and networks and the rural-urban food continuum across urban settlements in Kenya. Additional examination of rural-urban linkages and the potential of resource sharing and support systems between rural and urban communities is also warranted. Longitudinal studies may be beneficial in capturing the long-term effects of policy interventions and urban food environment changes. Broadening the scope of this study to include other urban settings and informal settlements across regions can yield a more comprehensive understanding of food security determinants in various contexts. Thus, the study enhances the understanding of factors influencing food security in informal urban settlements and contributes to the ongoing effort to address food insecurity challenges faced by urban households. Study insights can inform the development of more effective and targeted policies and interventions that would ultimately support SDG goals of achieving zero hunger and fostering sustainable urban development.

## Data availability statement

Data will be made available on request.

## Ethical approval and consent to participate in the study

The respondents involved in the study provided verbal consent before participating in the study. Moreover, all the research assistants involved in the study were trained on ethical procedures in data collection, including confidentiality.

## CRediT authorship contribution statement

**Oscar Ingasia Ayuya:** Writing – review & editing, Writing – original draft, Software, Project administration, Methodology, Funding acquisition, Formal analysis, Data curation, Conceptualization.

## Declaration of competing interest

The author declare no financial interests or personal relationships which may be considered as potential competing interests.
